# Vicarious Neural Processing of Outcomes during Observational Learning

**DOI:** 10.1371/journal.pone.0073879

**Published:** 2013-09-05

**Authors:** Elisabetta Monfardini, Valeria Gazzola, Driss Boussaoud, Andrea Brovelli, Christian Keysers, Bruno Wicker

**Affiliations:** 1 INSERM, U1028; CNRS, UMR5292; Lyon Neuroscience Research Center, ImpAct Team, Lyon, France; 2 Institut de Médecine Environnementale, Paris, France; 3 University Medical Center Groningen, University of Groningen, Department of Neuroscience, BCN NeuroImaging Center, Groningen, The Netherlands; 4 Netherlands Institute for Neuroscience, Royal Netherlands Academy of Arts and Sciences, Amsterdam, The Netherlands; 5 Institut de Neuroscience des Systèmes, UMR 1106, INSERM, Aix-Marseille Université, Marseille, France; 6 Institut de Neurosciences de la Timone, CNRS & Aix-Marseille Université, UMR 7289, Marseille, France; 7 Integrative Neuroscience Laboratory, Physics Department, University of Buenos Aires, Capital Federal, Argentina; Università di Parma, Italy

## Abstract

Learning what behaviour is appropriate in a specific context by observing the actions of others and their outcomes is a key constituent of human cognition, because it saves time and energy and reduces exposure to potentially dangerous situations. Observational learning of associative rules relies on the ability to map the actions of others onto our own, process outcomes, and combine these sources of information. Here, we combined newly developed experimental tasks and functional magnetic resonance imaging (fMRI) to investigate the neural mechanisms that govern such observational learning. Results show that the neural systems involved in individual trial-and-error learning and in action observation and execution both participate in observational learning. In addition, we identified brain areas that specifically activate for others’ incorrect outcomes during learning in the posterior medial frontal cortex (pMFC), the anterior insula and the posterior superior temporal sulcus (pSTS).

## Introduction

The capacity to vicariously learn from others which action is most rewarding in a particular situation is one of the most basic forms of human social cognition [1]–[3]. Learning-by-observation (LeO) plays a crucial role in many adaptive behaviours such as foraging and predator avoidance [4] and it has been observed in several animal species including rats [5], dogs [6], pigeons [7] and monkeys [8]–[11]. LeO relies on multiple functions, including the ability to infer others’ intentions from action observation, process others’ action outcomes (i.e. successes and errors) and combine these sources of information to learn arbitrary stimulus-action-outcome associations that can later serve the selection of behaviours leading to desired outcomes.

During individual trial-and-error learning (TE), decades of research have uncovered a detailed mechanistic understanding of how learning to select the most rewarding action in response to a stimulus is governed by multiple reward-related signals. Reward prediction-error signals (i.e. the difference between obtained and expected rewards) are represented in the ventral striatum [12], [13] and ventral tegmental area [14]–[16]. fMRI activations correlating with the absolute value of prediction errors signals have been found in the dorsal striatum [17] and in the dorsal fronto-parietal network [18]; and first correct outcomes selectively activate the left dorsolateral prefrontal cortex in humans [18] and produce specific signals in anterior cingulate cortex in monkeys [19]. Such a detailed mechanistic understanding still lacks for LeO. Recent results suggest that LeO depends on observational action prediction-errors (i.e. the actual minus the predicted action of others) and observational outcome prediction-errors (i.e. the actual minus predicted outcome received by others) that selectively recruit the dorsolateral and ventromedial prefrontal cortex, respectively ([20] see also [21]). The relationship of these signals with those recruited during TE remains, however, poorly understood.

Observing the actions of others is known to vicariously recruit brain regions traditionally associated with action execution [22]–[24]. The network of brain regions common to action observation and execution in humans has been dubbed the putative mirror neuron system (pMNS) in analogy to the mirror neurons found in similar brain regions in monkeys [25], [26]. This pMNS includes the ventral and dorsal premotor cortex, the inferior parietal lobule and adjacent somatosensory areas, and the middle temporal gyrus (see [24] for review). Such vicarious motor activations in the pMNS and what we know about mirror neurons from animal studies provide a powerful conceptual framework to understand how observers can learn to reproduce the observed actions of others (see [27] for review). But during LeO, how do observers learn which of many observed and vicariously activated actions are most rewarding in response to a particular stimulus? Here we explore whether activations in the pMNS coexist with representations of the outcomes obtained by the observed agents to make such LeO possible. Specifically, we explore whether representations of the outcomes of others depend on the vicarious recruitment of the brain circuits normally involved in individual TE and/or whether such information triggers activity in regions not as involved during TE. To this aim, we scanned human participants using functional magnetic resonance imaging (fMRI) while learning stimulus-action-outcome associations either by TE (i.e. first hand) or LeO (i.e. vicariously). This allowed us to identify, and for the first time directly compare, the brain networks mediating the processing of errors and successes during individual and observational learning.

## Materials and Methods

### Subjects

Eighteen healthy, right-handed volunteers (7 males) participated in the study (mean age: 27.6±4.5 years), but one was discarded for technical problems and two based on their poor learning performance. Consequently, fifteen subjects were included into the analysis (6 males; mean age: 27.1±4.7 years). The subjects were screened to rule out medication use, history of neurological or psychiatric disorders, head trauma, substance abuse or other serious medical conditions. Written consent was obtained after the procedure had been fully explained. The study was approved by the Medical Ethical Commission (METc) of the University Medical Center Groningen (NL). Volunteers were paid for their participation.

### Task Design

The experiment was built as an event-related paradigm with nine experimental runs. Each run consisted of a single task, corresponding to one of the following experimental conditions: learning by trial-and-error (TE), learning-by-observation (LeO), pMNS localizer. The two learning conditions were repeated four times in order to increase the number of events per condition. The ordering of runs was randomized across subjects. Each task was explained to the subjects step by step before scanning.

#### Learning by trial-and-error (TE)

**Figure 1:**
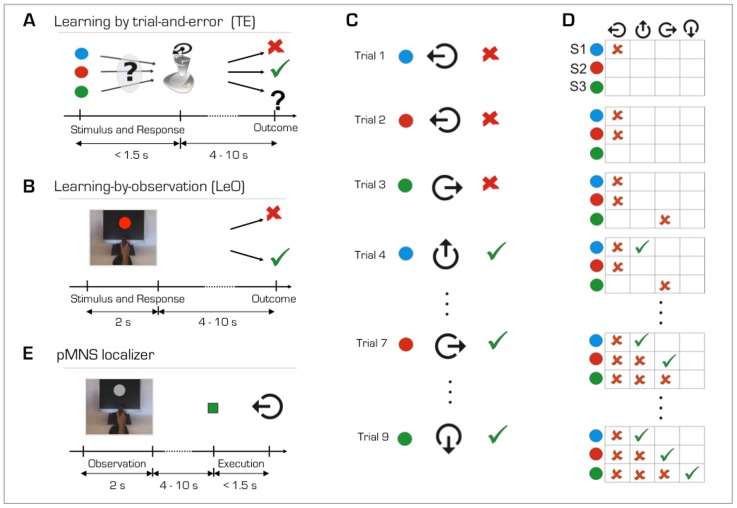
fMRI task design. (**A**) *Learning by trial-and-error* (TE). A trial started with the presentation of a coloured stimulus. Participants had to displace the joystick in one of the four possible directions (up, down, right and left) within 1.5 seconds. After a variable delay, a feedback stimulus was presented for 1 second indicating whether the action was correct (green tick), incorrect (red cross) or late (question mark). (**B**) *Learning-by-observation* (LeO). Each trial started with the presentations of a video showing a hand on a joystick performing one of the four possible movements in response to the presentation of a coloured stimulus on a monitor. The camera view was set to actor’s perspective. The video lasted 2 seconds and the coloured stimulus was presented for 1.5 seconds, as in the trial-and-error condition. The outcome images were presented after a variable delay and they were identical to those used in the TE condition. Participants were instructed to learn the correct stimulus-action-outcome associations by looking at the videos and outcomes. (**C**) Task design of an exemplar learning session. Stimuli were randomised in blocks of 3 trials. (**D**) Matrix of all possible stimulus-response combinations corresponding to the exemplar session in (C). Correct associations were not set *a priori*, but they were assigned as subjects advanced in the task. The first presentation of each stimulus was always followed by an incorrect outcome, irrespective of the motor response (from trial 1 to 3). On the second presentation of S1 (the blue circle), any untried joystick movement was always followed by a correct outcome (trial 4). The correct response for S2 and S3 (red and green circles, respectively) was found after 2 and 3 incorrect joystick movements (at trials 7 and 9, respectively). In other words, the correct response was the 2^nd^ joystick movement (different from the first tried response) for stimulus S1, the 3^rd^ joystick movement for stimulus S2, and the 4^th^ for stimulus S3. This task design ensured a minimum number of incorrect trials during acquisition (one for S1, two for S2 and three for S3) and fixed representative steps during learning. The LeO task was built using a design similar to the one used for the TE learning task. Given the scarcity of repetition and maintenance errors in TE, in LeO the actor neither repeated incorrect actions while searching for the correct association (i.e. no repetition errors in the acquisition phase of learning), nor made errors after the first correct response (i.e. no maintenance errors). Therefore, learning-by-observation consisted in 6 incorrect (one for S1, two for S2 and three for S3) and 12 correct trials. (**E**) Observation and execution of actions. Participants observed a video of a hand performing a joystick movement in response to a grey stimulus (i.e. action observation). After a variable delay, subjects were instructed to perform the movement they had previously observed (i.e. action execution).

During scanning, participants had to learn the correct associations between each of 3 coloured stimuli and 1 of 4 possible joystick movements (Fig. 1A). Subjects performed 4 TE learning sessions. To avoid confusion across runs, in each run, the coloured stimuli had a different geometric shape (e.g. triangles of 3 colours in one run, circles of 3 colours in another run, rhombus in another run, squares in another run still). On each trial, subjects were presented with a coloured shape and they had to make a decision within 1.5 s by moving the joystick in one of the 4 possible directions. After a variable delay ranging from 4 to 10 s (randomly drawn from a log-normal distribution) following the disappearance of the coloured stimulus, an outcome image was presented (Fig. 1A). The outcome image lasted 1s and informed the subject whether the response was correct (green tick-mark), incorrect (red-cross) or late (question-mark, if the reaction time exceeded 1.5s). In case of a late trial, the same visual stimulus was repeated in the next trial in order to obtain the same number of valid trials per session. Late trials (mean±standard error of the mean per subject: 1.62±0.26) were modeled at the first level of analysis with a predictor of no interest and thus excluded from the regressors of interest in later analyses. The next trial started after a variable delay ranging from 4 to 10s with the presentation of another visual stimulus. Visual stimuli were pseudo-randomized in blocks of three trials. Each learning session was composed of 18 trials, 3 stimulus types (i.e. identical shape but different colours, S1, S2, and S3) and 4 possible joystick movements. Thereafter, subject performed 12 trials in which they were tested on their knowledge of the associations (TE-test trials). In these trials, the stimuli appeared (1.5 s) on the screen and subjects were asked to perform the correct movement within 1.5 s. No feedback was presented to prevent improvement in performance.

In order to induce reproducible performances across runs and subjects, we adapted a task design previously developed by Brovelli et al. [27], [28] that ensures similar number of successful and unsuccessful attempts across learning sessions. In fact, the stimulus-response associations were not established *a priori*, but assigned as the subject progressed in the learning task (cf. legend of the Fig. 1C, D). Consequently, the task design ensured a minimum number of incorrect trials during acquisition (one for S1, two for S2 and three for S3) and fixed representative steps during learning.

#### Learning-by-observation task (LeO)

**Figure 2:**
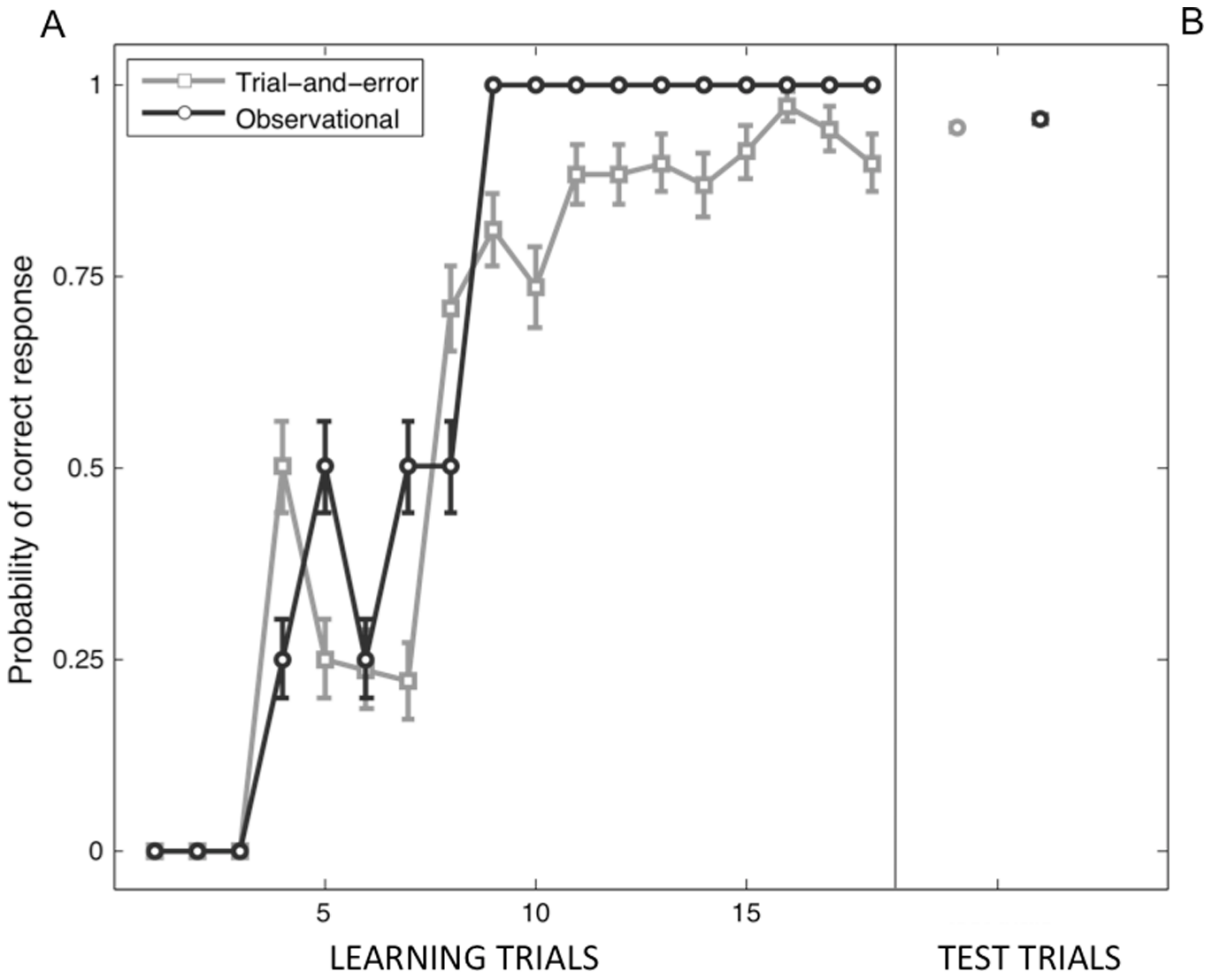
Behavioural performances of subjects in the fMRI learning sessions. (A) Mean learning curve averaged across runs and subjects for the TE condition (gray curve) and the LeO condition (black curve). Note that the LeO curve represents the progression of the actor performance in the videos shown to the participant. Error bars indicate the standard error of the mean (SEM). (B) Mean percentage of correct responses in the TE-test and LeO-test sessions following learning. Error bars indicate the standard error of the mean (SEM).

The LeO task was built using a similar task design. Subjects were asked to learn the associations between stimuli and joystick movements by observation of a video showing an actor learning the associations (Fig. 1B). The video lasted 2 seconds but the coloured stimulus was presented for 1.5 seconds, as in the TE condition, to make the timing of the conditions identical. After a variable delay ranging from 4 to 10s a positive or negative feedback appeared on the screen to inform whether the actor’s action was correct or incorrect. The subjects were instructed to learn the correct stimulus-response associations via the observation of the movies and the outcomes given to the actor. To ensure that both learning conditions contained similar numbers of successful and unsuccessful attempts, the progression of the actor performance was comparable to the actual performances of the subjects in the TE condition (Figure 1C, D). The actor never repeated the same incorrect action while searching for the correct association with a given stimulus (i.e. no repetition errors in the early phases of learning) and never made errors after the correct response (i.e. no maintenance errors). Each LeO session was composed of 18 learning trials as described above, 6 of which contained error trials. Visual stimuli were pseudo-randomized in blocks of three trials, except for the last trial of the third block (i.e 9^th^) that was always correct (this explains why the actor reached the 100% of correct responses on the trial 9, cf. Fig. 2A). Thereafter, subjects performed 12 trials in which they were tested on their knowledge of the associations (LeO-test trials). As in the TE-test trials, the outcome was not presented.

#### pMNS localizer task

This task was created to functionally map the brain areas activated during both action observation and execution, irrespectively of learning (Fig. 1E). To map action observation, subjects observed the movies (2s) used in the LeO condition to guarantee comparable visual characteristics across conditions. The colour of the visual stimulus was masked to remove the possibility to implicitly learn a visuomotor association. Participants were instructed to observe the action with the intent to repeat it. After a variable delay ranging from 4 to 10 s, a go signal (a green-cue) appeared on the screen to instruct the participants to execute the movement performed by the actor in the video. The execution phase lasted 1.5 s. Subjects saw 72 videos and therefore executed 72 actions in a single fMRI run.

#### Experimental set-up

Visual stimuli were projected at the centre of a screen positioned at the back of the scanner. Subjects could see the image reflected on a mirror (15×9 cm) suspended 10 cm in front of their faces and subtending visual angles of 42° horizontally and 32° vertically. The subject’s responses were recorded using an fMRI-compatible joystick (fORP, CurrentDesigns, Inc., Philadelphia, USA). Before the experiment, the participants were instructed that the correct stimulus-response associations were: (i) completely arbitrary, and (ii) not mutually exclusive (all stimuli could be associated with the same joystick movement), meaning that the subjects could not infer correct associations by excluding previous correct movements.

### fMRI Data Acquisition and Preprocessing

Images were acquired using a Philips Intera 3T Quaser, a synergy SENSE head coil, 30 mT/m gradients and a standard single shot-EPI with TE = 30 ms, TR = 2s, 37 axial slices of 3 mm thickness, with no slice gap and a 3×3 mm in plane resolution acquired to cover the entire brain and cerebellum. The slices were acquired in an interleaved spatial order. The first three volumes of each participant’s data were discarded to allow for longitudinal relaxation time equilibration.

Data were preprocessed with SPM5 (Wellcome Trust center for NeuroImaging, London, UK; http://www.fil.oin.ucl.ac.uk/software/spm5/). EPI images from all sessions were slice-time corrected and aligned to the first volume of the first session of scanning to correct for head movement between scans. A mean image was created using the realigned volumes. T1-weighted structural images were first co-registered to the mean EPI image of each participant. Normalization parameters between the co-registered T1 and the standard MNI T1 template were then calculated, and applied to the anatomy and all EPI volumes. Data were then smoothed using a 8 mm full-width-at-half-maximum isotropic Gaussian kernel to accommodate inter-subject differences in anatomy.

### fMRI Data Analysis

The statistical analysis of the pre-processed event-related BOLD signals was performed using a general linear model (GLM) approach. Each trial in the TE and LeO conditions consisted of two events. The first (SR: stimulus+response) was associated with the processing of the stimuli and the selection of motor response (TE), or the observation of movies (LeO). The second was associated with the processing of outcomes (O). To dissociate the two events in each trial, the regressors were constructed by convolving the canonical hemodynamic response function with delta functions of constant or varying amplitudes aligned on the time of SR and O onsets. Given that learning of stimulus-action-outcome associations only happens in the outcome phase, we only present the results related to brain activations recruited during the processing of outcomes (O). For the pMNS localizer trials, one regressor was aligned with the onset of videos presentation (action observation), the other with the go-cue onset (action execution).

#### Single participant analyses

The goal of the GLM analyses was to identify the cerebral networks involved in the processing of outcomes that displayed learning-related changes during TE and LeO. To do so, we computed two design matrices at the 1^st^ level of analysis. In the first one, the design matrix contained 10 regressors. The first 4 regressors modelled the BOLD responses in the TE condition. The 1^st^ and the 2^nd^ regressors were aligned on the stimulus presentation and included the trials in the acquisition (TE_SR_acquisition) and early consolidation (TE_SR_consolidation) phases of learning, respectively. The acquisition phase included the incorrect and 1st correct trial (Fig. 1C, D). The early consolidation phase was composed of all the trials starting with the second correct. The 3^rd^ and the 4^th^ regressors modelled the same learning phases, but they were aligned on the presentation of the outcome image (TE_O_acquisition and TE_O_consolidation, respectively). The same trial and event types in the LeO condition were modelled from the 5^th^ to the 8^th^ regressors (LeO_SR_acquisition, LeO_SR_consolidation, LeO_O_acquisition and LeO_O_consolidation). The 9^th^ and 10^th^ regressors included the trials of the action observation and execution (pMNS localizer).

In a second GLM, we refined the first analysis to dissociate the neural systems associated with processing of incorrect and first correct trials. We thus created a design matrix at 1^st^ level that contained 12 regressors (6 regressors for both TE and LeO). For each learning condition, three regressors were aligned on the SR event and three on O. Among these, the first regressor included incorrect trials (TE_SR_incorrect, TE_O_incorrect; LeO_SR_incorrect, LeO_O_incorrect), the second included the first correct trial for each association (TE_SR_1^st^correct, TE_O_1^st^correct; LeO_SR_1^st^correct, LeO_O_1^st^correct), whereas the third included subsequent correct trials (TE_SR_consolidation, TE_O_consolidation; LeO_SR_consolidation, LeO_O_consolidation).

#### Group analyses

All the fMRI statistics and P values arise from group random-effects analyses on the outcome phase of learning. Group analyses were thresholded at the voxel-level at *p*<0.001(uncorrected). The minimum cluster size (k) was 15 voxels, which ensured a cluster *p*≤0.05. To control the overall rate of false positives and because we searched for significant effects over the entire brain, we only report (unless specified otherwise) results with a False Discovery Rate (FDR) *q*<0.05 (k = 15 voxels).

The brain regions recruited during the acquisition phase of learning in both TE and LeO conditions were mapped using a two-way repeated-measures ANOVA, with 2 learning phases (acquisition and consolidation) × 2 learning conditions (TE and LeO). The learning signals are mainly processed in the acquisition phase, and voxels processing these signals should thus show an effect of phase, with the acquisition phase showing more activation than the consolidation phase. Such an effect dissociates processes associated with early learning (i.e. acquisition) from the sensory processing of the outcome (i.e. consolidation). If this effect is a main effect, without significant interaction with learning condition, the voxel would be similarly involved in learning for TE and LeO. If the voxel additionally shows an interaction with learning condition, it would be evidence of its stronger involvement in one form of learning than in the other.

To refine this analysis, an additional two-way repeated-measures ANOVA was implemented, with 2 correctness (incorrect, 1^st^correct) × 2 learning conditions (TE, LeO). The goal of this analysis was to dissociate the neural systems relative to the processing of incorrect and first correct trials during TE and LeO learning conditions.

Finally, we mapped the pMNS by first running two one-sample t-tests on the single participant beta values from the action observation and execution conditions. The thresholded group t-map resulting from the conjunction analysis [29] between observation and execution regressors (t = 3.41, *p*unc<0.001) was used as a localizer mask for the pMNS.

The anatomical location of each activated cluster was assessed using the SPM anatomy toolbox (https://www.fz-juelich.de/ime/spm_anatomy_toolbox) [30] and the Talairach Daemon software (http://www.talairach.org) [31]. Graphical display was performed using MRIcron software (http://www.cabiatl.com/mricro/mricron/index.html).

**Figure 3:**
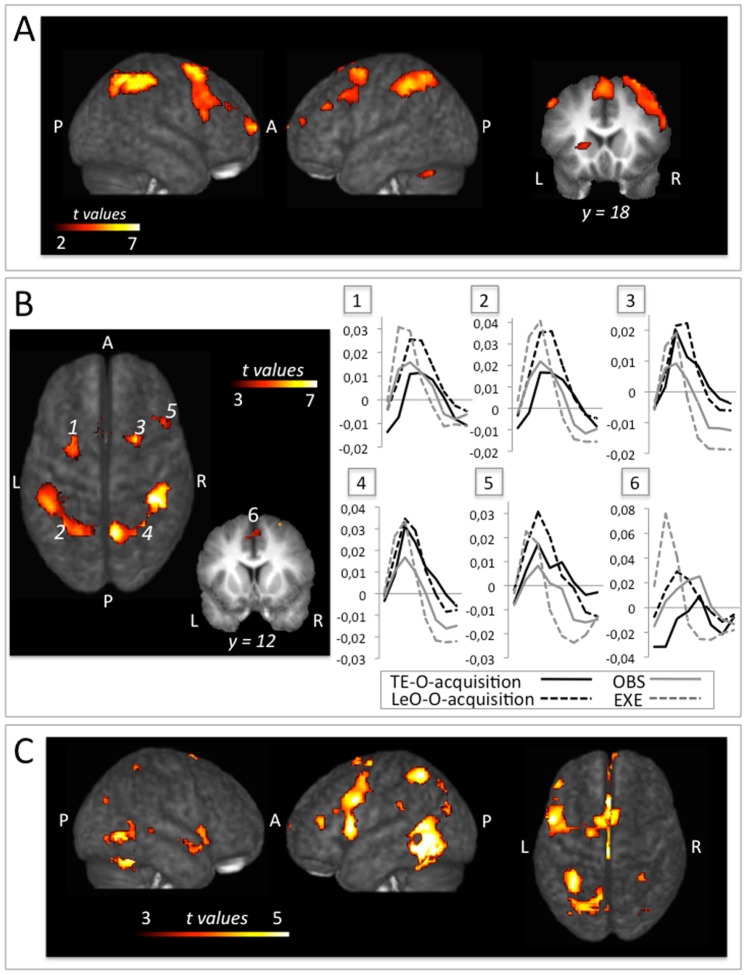
Clusters of activation are superimposed on to the average T1 image derived from all participants. (A) Brain networks commonly recruited during the acquisition phase of learning (i.e. incorrect trials +1^st^ correct trial) in both TE and LeO. Active brain regions in both TE (i.e. TE_O_acquisition>TE_O_consolidation) and LeO (i.e. LeO_O_acquisition>LeO_O_consolidation) contrasts (conjunction thresholded at *p*unc<0.001, t = 3.24;k = 15; all clusters also survive *q*FDR<0.05). See also Fig. S1. (B) Brain networks commonly recruited during the acquisition phase of learning, action observation and execution. Intersection analysis between the results from (A) and the localizer mask for the pMNS. Grand-average BOLD responses in the regions of overlap for TE_O_acquisition and LeO_O_acquisition (black and gray continuous line), OBS and EXE (continuous and dotted light gray) conditions. (C) Brain networks commonly recruited during the processing of 1^st^ correct outcome in TE and LeO. Positive effect of the 1st correct outcome (LeO_O_1stCorrect+TE_O_1stCorrect-LeO_O_incorrect-TE_O_incorrect) exclusively masked with the interaction of correcteness by learning condition (t = 3.24; *p*unc<0.001, k = 15; all clusters also survive *q*FDR<0.05).

**Figure 4:**
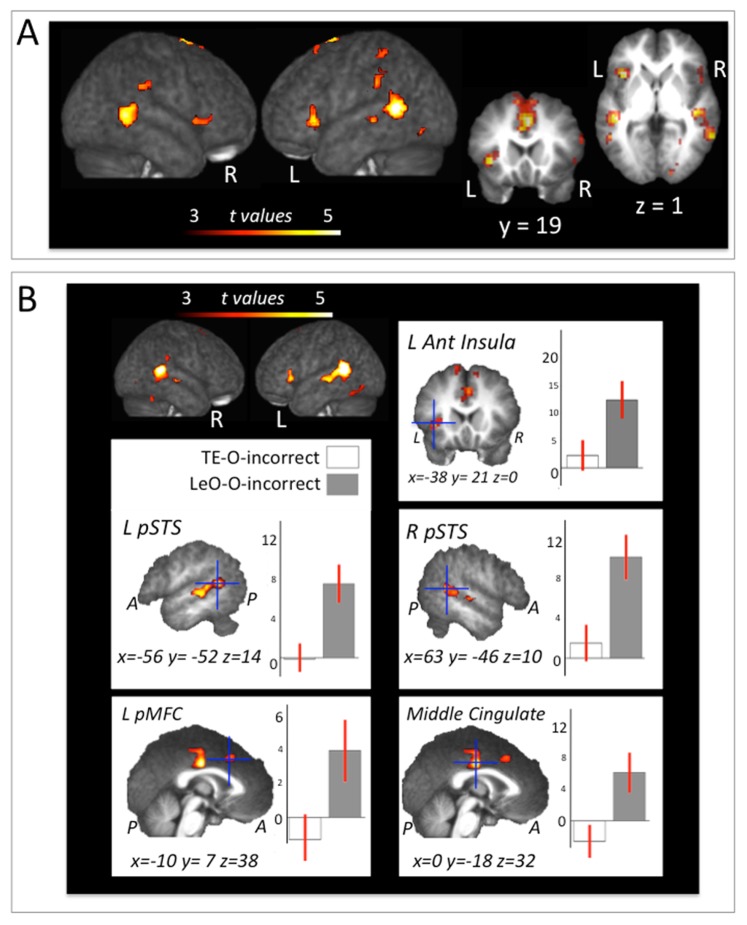
Clusters of activation are superimposed on the average T1 image derived from all participants. (A) **Direct comparison between LeO_O_acquisition and TE_O_acquisition.** Results from Leo_O_acquisition>TE_O_acquisition t-contrast (t = 3.24; *p*unc<0.001, k = 15; all clusters also survive *q*FDR<0.05). (B) **Direct comparison between LeO_O_incorrect and TE_O_incorrect.** Areas showing greater activation for processing of incorrect outcomes in LeO, with respect on processing of incorrect outcomes in TE (*p*unc<0.001, k = 15; all clusters also survive *q*FDR<0.05). Plot of the mean value of the parameter estimates (arbitrary units) for the maxima of the left anterior insula, left and right pSTS, left pMFC and middle cingulate cortex.

To depict the BOLD dynamics across conditions, we extracted the BOLD responses from all voxels in each activated cluster using the MarsBar toolbox for SPM (http://marsbar.sourceforge.net/). The average BOLD response was calculated by temporally aligning the BOLD time series on outcome onset and by averaging them across trials and subjects for each experimental condition (TE, LeO, OBS and EXE; Fig. 3B). Two separate three-way repeated measures ANOVAs with 2 conditions (observing others vs doing) × 2 tasks (learning vs. not learning) × 6 ROIs were then conducted by considering both (i) the peak values and (ii) the Δ scores (peak value-first point) of the curves shown in the Fig. 3B. In addition, we also plotted the mean value of the parameter estimates for the maxima of each clusters (Fig. 4B).

## Results

### Behaviour

In order to compare the neural substrates of trial-and-error and observational learning, we used a task designed to induce comparable performances across sessions, subjects and learning conditions (Fig. 1). Indeed, the mean learning curves, averaged across runs and subjects for the TE condition (Fig. 2A, gray curve), showed a profile comparable to the learning profile in the LeO condition (Fig. 2A, black curve; *r* = 0.55). The number of repetition and maintenance errors in the TE condition was very limited (mean±standard error of the mean per subject: *repetition errors* 0.78±0.16; *maintenance errors* 0.6±0.12). In addition, the mean percentage of correct responses in the test sessions following learning was 94.01% ±0.58% (mean+/− standard error of the mean) and 95.06% ±0.53% for the TE-test and LeO-test phases (*p* = 0.6; cf. Fig. 2B), respectively. Overall, the behavioural results showed that the task design successfully manipulated learning performance and induced reproducible performances across sessions and subjects. Most importantly, no significant difference was observed in the final performance after TE and LeO (Fig. 2B).

### Neuroimaging

#### Networks for the processing of outcomes during TE and LeO

A group-level 2×2 repeated-measures ANOVA with two phases (acquisition, consolidation) and two learning conditions (LeO, TE) showed a main effect of learning phase (F1,14 = 12.06, *p*unc<0.001; F1,14 = 6.47, *q*FDR<0.05), and a main effect of learning condition (F1,14 = 12.06, *p*unc<0.001; F1,14 = 9.81; all clusters also survive *q*FDR<0.05). The interaction of learning phase by learning condition (F1,14 = 12.06, *p*unc<0.001) was only significant in 23 voxels (13 voxels in left anterior insula extending to inferior frontal gyrus, 10 voxels in left posterior superior temporal sulcus) and therefore did not survive an FDR correction (*q*FDR = 0.35).

Post-hoc t-tests revealed that the activations specifically involved during the acquisition phase of learning (i.e. LeO_O_acquisition+TE_O_acquisition - LeO_O_consolidation - TE_O_consolidation) were localized in a large network of brain regions including the bilateral inferior (IPL, BA40) and superior (SPL, BA7) parietal cortex, the bilateral postcentral gyrus (BA2), the bilateral dorsal premotor (PMd, BA6) and dorsolateral prefrontal (dlPFC, BA9) cortices, the supplementary motor area (SMA), the bilateral middle temporal cortex (BA21/22), the bilateral cerebellum as well as the right caudate nucleus (dorsal striatum), the right inferior frontal gyrus (vlPFC, i.e. ventro-lateral prefrontal cortex, BA44/45/47) and the left anterior vlPFC (BA10) (t = 3.24, *p*unc<0.001, k = 15; all clusters also survive *q*FDR<0.05; cf. Fig. S1, Table S1). None of these regions showed an interaction of learning phase and condition. Consequently, this result suggests that the learning signal provided by outcomes yielded similar activations in these brain regions during the acquisition phase of both TE and LeO. To confirm the significance of the recruitment of this brain network in both TE and LeO acquisition phase individually, we also computed a conjunction analysis [29] between two contrasts: (TE_O_acquisition>TE_O_consolidation) ∩ (LeO_O_acquisition>LeO_O_consolidation). The results showed that outcome processing during TE and LeO acquisition phase commonly activated the bilateral IPL and SPL (BA40 and BA7, respectively), the bilateral PMd (BA6), the SMA, the bilateral cerebellum, the bilateral dlPFC (BA46/9), the right vlPFC (BA45/44), the left anterior vlPFC (BA10) and the left dorsal striatum (t = 3.24, *p*unc<0.001, k = 15; all clusters also survived *q*FDR<0.05; cf. Fig. 3A and Table 1A). These common brain activations, and the scarcity of voxels showing significant interactions, confirm that neural mechanisms engaged during acquisition LeO are strictly similar to those engaged during acquisition TE learning in humans.

**Table 1:** (A) Brain networks commonly recruited during the acquisition phase (i.e. incorrect outcomes+1^st^ correct outcome) in both TE and LeO conditions (conjunction thresholded at *p*unc<0.001, t = 3.24; k = 15; all clusters also survive *q*FDR<0.05).

Cluster size	Cluster P value (FWE corr)	Cluster P value (unc)	Peak MNI coordinates (x, y, z)	Peak P value (FDR-cor)	Peak T value	Brain Area	Hemisphere
*Table 1A*							
1257	0.000	0.000	42 −38 46	0.000	7.63	IPL (BA40, hIP2)	R
			38 −63 42	0.000	7.54	Angular Gyrus	R
			10 −66 56	0.000	7.33	SPL (7A)/Precuneus	R
374	0.000	0.000	32 7 66	0.000	7.12	PMd (SFG, BA6)	R
			42 21 42	0.000	4.99	dlPFC (MFG) (BA46/9)	R
			60 18 24	0.000	4.79	vlPFC (IFG, BA45/44)	R
242	0.000	0.000	0 24 46	0.000	6.05	SMA	R/L
			−4 10 56	0.000	5.65	SMA (BA6)	L
217	0.000	0.000	−24−4 52	0.000	5.93	PMd (SFG, BA6)	L
			−28 4 66	0.000	5.90	PMd (SFG, BA6)	L
			−49 18 42	0.000	5.09	PMd (MFG) (BA 6)	L
136	0.000	0.000	−28 −63 −35	0.000	5.54	Cerebellum (Lobule VIIa)	L
			−10 −77 −32	0.000	4.93	Cerebellum (Lobule VIIa)	L
			10 −77 −28	0.003	3.90	Cerebellum (Lobule VIIa)	R
78	0.001	0.000	35 −63 −32	0.000	6.51	Cerebellum (Lobule VIIa, Lobule VI)	R
			21 −60 −35	0.004	3.74	Cerebellum (Lobule VI)	R
59	0.005	0.001	28 66 10	0.000	5.66	Anterior vlPFC (SFG, BA10)	R
28	0.093	0.011	−42 35 32	0.000	5.73	dlPFC (MFG) (BA9)	L
17	0.293	0.040	−28 56 18	0.000	4.59	Anterior vlPFC (BA10)	L
17	0.293	0.040	−21 18 0	0.002	3.98	Dorsal striatum (Caudate)	L
*Table 1B*							
231	0.000	0.000	−42 −46 52	0.003	5.73	BA2/hIP2	L
			−46 −42 42	0.005	5.47	IPL (hIP2)	L
			−32 −60 60	0.008	5.45	SPL (BA7A)	L
211	0.000	0.000	42 −38 46	0.000	7.63	IPL, Postcentral Gyrus (BA40/BA2)	R
			10 −66 56	0.000	7.33	Precuneus	R
			35 −60 60	0.002	6.06	SPL (BA 7A)	R
63	0.004	0.000	−28 4 66	0.002	5.90	PMd (SFG, BA6)	L
			−24 −4 56	0.008	5.32	PMd (SFG, BA6)	L
39	0.032	0.004	32 7 66	0.000	7.12	PMd (SFG, BA6)	R
28	0.093	0.011	42 21 42	0.019	4.99	PMd (MFG, BA6)	R
			49 18 38	0.034	4.76	PMv (IFG p. Opercularis, BA44)	R
			56 21 35	0.052	4.67	PMv (IFG p. Opercularis, BA44)	R
19	0.237	0.031	4 21 46	0.052	4.69	SMA	R
			0 10 52	0.113	4.31	SMA	R/L
*Table 1C*							
3561	0.000	0.000	−46 −56 −4	0.000	7.23	MTG (BA21)	L
			32 −38 −21	0.000	6.87	Fusiform Gyrus	R
			−46 −56 −18	0.000	6.72	Fusiform Gyrus	L
686	0.000	0.000	−4 −28 32	0.000	5.91	Middle Cingulate (BA23)	L
			−49 4 35	0.000	5.32	dlPFC (BA9)	L
			−49 10 7	0.000	5.27	IFG (p. Opercularis, BA44)	L
			4 21 28	0.000	5.17	Anterior Cingulate Cortex (BA24,32)	R
182	0.000	0.000	−32 −60 56	0.000	5.13	SPL (7A)	L
			−28 −52 52	0.000	4.93	SPL (7PC)	L
			−32 −38 49	0.000	4.57	Postcentral Gyrus (BA2)	L
45	0.028	0.004	4 66 7	0.001	4.10	SMG (mPFC, BA10)	R
			4 52 4	0.001	3.96	SMG (mPFC, BA10)	R
			0 42 7	0.003	3.51	Anterior Cingulate (ACC)	R/L
33	0.077	0.011	32 −80 28	0.002	3.85	Middle Occipital (BA19)	R
			42 −70 28	0.002	3.78	Middle Occipital (BA19)	R
29	0.110	0.016	−46 38 14	0.001	4.30	IFG (p. Triangularis, BA45)	L
18	0.305	0.048	−52 −38 28	0.002	3.90	SupraMarginal Gyrus (BA40)	L

See also Table S1. **(B)** Brain networks commonly recruited during learning acquisition phase, action observation and execution. Intersection analysis thresholded at *p*unc<0.001 (all clusters also survive *q*FDR<0.05). **(C)** Brain networks commonly recruited during the processing of 1^st^ correct outcome in TE and LeO. Positive effect of the 1st correct outcome (LeO_O_1stCorrect+TE_O_1stCorrect-LeO_O_incorrect-TE_O_incorrect) exclusively masked with the interaction of correcteness by learning condition (t = 3.24; *p*unc<0.001, k = 15; all clusters also survive *q*FDR<0.05). Abbreviations: IPL (inferior parietal lobule); SPL (superior parietal lobule); IFG (inferior frontal gyrus); SFG (superior frontal gyrus); MFG (middle frontal gyrus); SMA (supplementary motor cortex); STG (middle temporal gyrus); MTG (middle temporal gyrus); IFG (inferior frontal gyrus); vlPFC (ventro-lateral prefrontal cortex); dlPFC (dorso-lateral prefrontal cortex); PMd (premotor dorsal); PMv (premotor ventral); BA: Brodmann area.

To further explore the effect of learning type, we investigated the difference between LeO and TE during the acquisition phase of learning. The contrast TE_O_acquisition>LeO_O_acquisition revealed no significant clusters (*p*unc<0.001, *q*FDR>0.149). The opposite contrast (LeO_O_acquisition>TE_O_acquisition), revealed BOLD changes reflecting specific LeO-related activity in middle and anterior cingulate gyri extending to SMA, in bilateral posterior superior temporal sulcus (pSTS), left anterior insula (BA13), bilateral supramarginal gyrus (BA40), bilateral fusiform gyrus and in left inferior frontal gyrus (BA44/45; t = 3.24, *p*unc<0.001; all clusters survived *q*FDR<0.05; cf. Fig. 4A and Table 2A).

**Table 2:** Direct comparison between LeO_O_incorrect and TE_O_incorrect.

Cluster size	Cluster P value (FWE corr)	Cluster P value (unc)	Peak MNI coordinates (x, y, z)	Peak P value (FDR-cor)	Peak T value	Brain Area	Hemisphere
*Table 2A*							
444	0.000	0.000	0 −18 35	0.141	5.02	Middle Cingulate (BA24)	L/R
			4 18 38	0.141	4.98	Anterior Cingulate/pMFC (BA24/32)	R
			10 14 60	0.175	4.80	SMA (BA6)	R
156	0.000	0.000	−56 −56 10	0.094	5.67	STG (BA22)/pSTS	L
			−46 −32 −4	0.141	5.04	MTG (BA21)	L
			−38 −42 −14	0.319	4.45	ITG (BA20)	L
152	0.000	0.000	49 −24 −4	0.141	4.97	STG (BA22)	R
			63 −52 4	0.175	4.84	MTG (BA21)/pSTS	R
			60 −35 4	0.783	3.57	MTG (BA21)	R
78	0.001	0.000	−38 21 0	0.141	5.24	Anterior inferior Insula (BA13/14)	L
			−28 10 4	0.423	4.08	Anterior inferior Insula (BA13/14)	L
46	0.017	0.002	−49 −32 24	0.306	4.50	SupraMarginal Gyrus (IPL, BA40)	L
			−52 −35 32	0.423	4.09	SupraMarginal Gyrus (IPL, BA40)	L
			−42 −21 14	0.632	3.72	Rolandic Operculum	L
46	0.017	0.002	28 −49 −10	0.354	4.24	Fusiform Gyrus	R
			28 −56 −4	0.787	3.55	Lingual Gyrus (BA17)	R
45	0.018	0.002	28 −77 −7	0.325	4.37	Fusiform Gyrus	R
			18 −94 0	0.496	3.90	Calcarine Gyrus (BA17)	R
21	0.192	0.025	−38 −77 −14	0.496	3.93	Fusiform Gyrus	L
19	0.237	0.031	63 −35 32	0.531	3.84	SupraMarginal Gyrus (IPL, BA40)	R
19	0.237	0.031	−35 −42 60	0.713	3.65	Postcentral Gyrus (BA2)	L
16	0.325	0.046	52 14 0	0.711	3.67	IFG (p. Opercularis, BA44)	R
			52 28 4	0.913	3.37	IFG (p. Triangularis, BA45)	R
*Table 2B*							
229	0.000	0.000	−56 −52 14	0.001	6.16	STG (BA22)/pSTS	L
			−46 −32 −4	0.003	4.78	MTG (BA21)	L
			−38 −42 −14	0.012	3.90	ITG (BA20)	L
224	0.000	0.000	63 −46 10	0.001	5.94	MTG (BA21)/pSTS	R
			49 −24 −4	0.003	4.97	STG (BA22)	R
			32 −24 7	0.005	4.52	Insula (Ig1)	R
200	0.000	0.000	0 −18 32	0.001	5.34	Middle Cingulate (BA24)	R/L
			10 −28 46	0.004	4.73	Middle Cingulate (BA24)	R
			−4 −21 46	0.007	4.28	Middle Cingulate (BA24)	L
107	0.000	0.000	28 −80 −7	0.006	4.47	Fusiform Gyrus (hOC3v)	R
			35 −63 −21	0.010	4.03	Cerebellum	R
			38 −70 0	0.011	3.95	MOG (BA19)	R
83	0.002	0.000	−28 10 4	0.003	4.83	Anterior inferior Insula (BA 13/14)	L
			−38 21 0	0.004	4.67	Anterior inferior Insula (BA 13/14)	L
			−21 0 7	0.018	3.54	Putamen	L
71	0.004	0.001	−10 7 38	0.006	4.41	pMFC (BA 32)	L
			4 14 38	0.009	4.11	pMFC (BA 32)	R
			−7 24 28	0.011	3.96	pMFC (BA 32)	L
54	0.014	0.002	−38 −60 −21	0.013	3.86	Fusiform Gyrus	L
			−32 −77 −10	0.014	3.75	IOG	L
49	0.020	0.003	28 −49 −10	0.004	4.74	Fusiform Gyrus	R
36	0.060	0.008	10 14 60	0.006	4.37	SMA (BA6)	R
			14 0 63	0.017	3.56	SMA (BA6)	R
18	0.305	0.048	28 −35 56	0.012	3.92	Precentral Gyrus (BA 3)	R
17	0.335	0.054	28 −21 60	0.013	3.86	Precentral Gyrus (BA 6)	R

**(A)** Results from Leo-O-acquisition>TE-O-acquisition t-contrast (t = 3.24; *p*unc<0.001, k = 15; all clusters also survive *q*FDR<0.05). **(B)** Areas showing greater activation for processing of outcome in LeO, with respect on processing of outcome in TE (*p*unc<0.001, k = 15; all clusters also survive *q*FDR<0.05). Abbreviations as in Table1. pMFC (posterior Medial Frontal Cortex); pSTS (posterior Superior Temporal Sulcus); MOG (middle occipital gyrus); IOG (inferior occipital gyrus).

#### Processing of outcomes and the putative Mirror Neuron System

In order to investigate whether the pMNS is activated when the outcome is revealed during the acquisition phase, we acquired a pMNS localizer (t = 3.4, *p*unc<0.001; cf. Table S2), which identified the key parietal (BA2/PF/PFop and intraparietal sulcus hIP2) and premotor (PMv, PMd, SMA) regions consistently associated with the pMNS [23], [24]. We then inclusively intersected the pMNS localizer with the activations common to TE and LeO during the acquisition phase of learning [(TE_O_acquisition>TE_O_consolidation) ∩ (LeO_O_acquisition>LeO_O_consolidation)]. As shown in Fig. 3B and Table 1B, overlap analysis between learning-related network and pMNS revealed clusters in the bilateral superior (BA7A) and inferior (PF/PFop, hIP2) parietal lobes, the postcentral gyrus (BA2), in the bilateral PMd (BA6), in the ventral premotor cortex (right inferior frontal gyrus, BA44) and in the SMA (Fig. S2, Table S3). Averaging the time courses of BOLD response relative to the time at which the outcome is revealed is illustrated in Fig. 3B and shows a distinctive peak of activity after the outcome in all the clusters. Three-way repeated measures ANOVAs with 2 conditions (observing others vs. doing) ×2 tasks (learning vs. not learning)×6 clusters revealed no significant three way interaction (condition× task× cluster, Δ scores: F5,84 = 1.36, p = 0.25; peak values: F5,84 = 1.46, p = 0.21), meaning that similar patterns of BOLD signal change were found across the different region of interest (ROI). However, the interaction effect of condition× task revealed significant results (Δ scores: F1,84 = 13.36, p<0.001; pick values: F1,84 = 68.64, p<0.001), which suggests that the BOLD activity in all six ROIs showed a different effect of condition depending on task. In other words, the BOLD activity in these areas presented an opposite pattern depending on whether the subjects were involved in a learning task or not.

#### Networks for the processing of incorrect and 1^st^ correct outcomes during trial-and-error and learning-by-observation

This analysis was aimed to refine the understanding of neural dynamics engaged during acquisition phases of learning by differentiating the processing of errors and 1^st^ correct trials in both TE and LeO conditions. A two-way repeated-measures ANOVA with 2 correctness (incorrect, 1^st^correct) × 2 learning conditions (TE, LeO) revealed a main effect of correctness (F1,14 = 12.06, *p*unc<0.001; F1,14 = 5.89, *q*FDR<0.05); a main effect of learning condition (F1,14 = 12.06, *p*unc <0.001; F1,14 = 12.17, all clusters also survive *q*FDR<0.05); and a trend toward an interaction of correcteness by learning condition (F1,14 = 12.06, *p*unc<0.001 but *q = *NS; see Table S4). The contrast LeO_O_incorrect+TE_O_incorrect-LeO_O_1stCorrect-TE_O_1stCorrect yielded no significant voxels (*p*unc<0.01, *q*FDR>0.99), while the opposite contrast LeO_O_1stCorrect+TE_O_1stCorrect-LeO_O_incorrect-TE_O_incorrect revealed significant BOLD increases (t = 3.24, *p*unc<0.001; all clusters also survive *q*FDR<0.05).

Before interpreting voxels as being equally modulated by correctness in both learning conditions, we identified voxels showing an interaction between correctness and learning type. Although this interaction was not significant using an FDR correction, it was significant at an uncorrected threshold in some voxels (F1,14 = 12.06, *p*unc<0.001; see Table S4). To isolate the brain regions that showed a similar preference for 1^st^ correct over incorrect trials for LeO and TE, we therefore exclusively masked the results of positive effect of 1^st^ correct trials with the interaction effect of learning type by correctness (F1,14 = 12.06, *p*unc<0.001). The results revealed BOLD changes bilaterally in the fusiform gyrus, in the left middle temporal gyrus (BA21) and in the middle and anterior cingulate gyrus extending to the left dlPFC (BA9) and vlPFC (BA44/45), as well as in the left SPL (BA7), left postcentral gyrus (BA2), left supramarginal gyrus (BA40), right superior medial gyrus (BA10) and right middle occipital gyrus (BA19; Fig. 3C and Table 1C). The current result suggests that the processing of 1^st^ correct outcomes has a crucial role for both TE and LeO and relies on similar neural computations.

We calculated the following contrasts to explore differences in the processing of *1^st^correct outcomes* in TE and LeO: LeO_O_1^st^correct>TE_O_1^st^correct and TE-O-1^st^correct>LeO_O_1^st^correct contrasts. In both cases, no brain areas displayed differences that survived our thresholds (i.e. *p*unc<0.001 and *q*FDR<0.05).

Finally, two t-contrasts were calculated in order to examine whether particular brain regions might be specifically involved in the processing of *errors* in one of the learning conditions: 1) LeO_O_incorrect>TE_O_incorrect; 2) TE_O_incorrect>LeO_O_incorrect. The contrast LeO_O_incorrect>TE_O_incorrect revealed a network of brain areas showing significantly greater activation when the subject processed other’s errors. This network includes the left medial temporal and the bilateral superior temporal gyrus (respectively, BA21 and BA22) including the posterior superior temporal sulci (pSTS), the bilateral anterior insula and the middle and anterior cingulate gyrus (BA32, BA24) encompassing the posterior medial frontal cortex (pMFC) (t = 3.24, *p*unc<0.001; all clusters also survive at *q*FDR<0.05; cf. Fig. 4B, Table 2B). Other clusters were identified in the bilateral fusiform gyrus extending to the cerebellum on the right hemisphere, in the SMA and in the right postcentral gyrus (BA3). No brain areas were found in the opposite contrast (TE_O_incorrect>LeO_O_incorrect), neither at *p*unc<0.001 nor at *q*FDR<0.05.

## Discussion

The aim of the current study was to explore the neural substrates allowing us to learn the correct action to perform in a particular situation by observing the successes and failures of others. We investigated the neural systems involved in the processing of others' successes and errors during learning-by-observation (LeO), and compared them to those recruited during trial-and-error (TE) learning. The experimental learning tasks were designed to produce reproducible phases of acquisition and consolidation across sessions and individuals during LeO and TE. This allowed us to compare brain activations across learning types at different stages of learning, from acquisition to early consolidation. In addition, we investigated the role of the pMNS during learning by mapping brain areas involved in both action observation and action execution.

### Common Brain Networks Mediating Individual and Observational Learning

Our study shows that, independently of whether learning is achieved by observation or trial-and-error, the processing of outcomes during acquisition (as compared with early consolidation) is mediated by brain regions encompassing three documented cerebral systems: the dorsal fronto-parietal, the fronto-striatal, and the cerebellar networks. These brain systems are activated during both TE and LeO (Fig. 3A, Table 1A; see also Fig. S1 and Table S1), and display stronger activation during the initial learning phase, when outcomes drive learning signals, than during the following correct trials in the early consolidation phase. The dorsal fronto-parietal system, which comprises the superior and inferior parietal lobes and the premotor dorsal cortex bilaterally, is thought to play a key role in sensorimotor transformation [32], [33], in the control of goal-directed attention to salient stimuli and responses [34], and in instrumental learning (e.g. [35], [36]). Previous neuroimaging studies have also confirmed its role in trial-and-error learning [37]–[39] and more specifically in the processing of outcomes [18]. This suggests that the processing of others' successes and errors during LeO partly exploits the same neural system mediating individual learning, visuomotor transformations and the control of goal-direct attention.

Our fronto-striatal network comprises the left dorsal striatum, the anterior ventro-lateral, dorso-lateral prefrontal cortices and the SMA. These structures form the associative fronto-striatal loop thought to subserve goal-directed processes during individual instrumental learning [40]–[47]. Previous work has shown learning-related activities during individual learning in the head of the caudate nucleus and portions of the prefrontal cortex (ventrolateral and dorsolateral), as well as in the premotor and supplementary motor areas [17], [18], [48]–[55]. In particular, the anterior caudate nucleus may integrate information about performance and cognitive control demands during individual instrumental learning [28], whereas the ventrolateral prefrontal cortex is implicated in the retrieval of visuomotor associations learned either by trial-and-error or by observation of others' actions [56]. Again, the overlap in the fronto-striatal network of learning specific activity during LeO and TE suggests that the processing of outcomes during observational learning relies, additionally to the dorsal fronto-parietal system, on a fronto-striatal network that is pivotal for individual instrumental learning. During TE, this system is thought to create an association between actions and outcomes. We suggest that during LeO, the same network encodes associations between the vicariously represented actions of others and their outcomes, which can later be used to guide the observers’ own behaviour.

The last network involved in outcome processing during the early phases of TE learning is located bilaterally in the cerebellum. Clinical reports on cerebellar patients describe severe impairments in cognitive planning and procedural learning (e.g. [57]–[60]). Moreover, using repetitive transcranial magnetic stimulation (rTMS), Torriero et al. [61] provided evidence in favour of a role of cerebellar structures during the acquisition of new motor patterns both by-observation and trial-and-error. The activation of the cerebellum in our study suggests that this structure is involved in both TE and LeO, even when new motor patterns do not need to be learned.

The fact that LeO depends in part on the brain mechanisms of TE is further supported by the observation that a common network of brain areas is also engaged during the processing of first correct outcomes in both learning processes. Previous studies examining TE learning found a selective increase in activity in the dorsolateral prefrontal cortex, BA9 [18] on first correct trial and the inferior frontal gyrus has been shown to selectively activate on first correct trial [38]. Such selective activation upon first correct outcomes may be responsible for our ability to rapidly learn stimulus-response-outcome associations. The selective activation at first correct outcomes during LeO, as revealed by our study, suggests that the dorsolateral prefrontal cortex is involved in rapid, seemingly one-trial, learning, irrespectively of the type of learning mechanisms (through trial-and-error or observation). Alternatively, this activation may allow the correct implementation of learning strategies such as the repeat-stay (perform the same action if previously rewarded). These interpretations are in line with previous reports showing deficits in rapid arbitrary visuomotor learning and strategy use after lesions of the lateral and orbital prefrontal cortex [62] and electrophysiological findings showing a selectivity in the discharge of prefrontal neurons for the type of learning strategy [63].

Taken together, our results suggest that the processing of other's outcomes during the acquisition of visuomotor associations by observation is largely implemented by a neural circuit overlapping with the brain areas involved in individual trial-and-error learning.

### Role of the Putative Mirror Neuron System During Learning-by-observation

Previous research on observational learning in humans has focused on the acquisition of novel motor patterns through imitational and mirror-like mechanisms. In these tasks, participants do not need to choose amongst multiple observed actions. Instead, they have to imitate observed actions, without any of the actions leading to positive or negative outcomes. Several of these studies have reported that the fronto-parietal pMNS is strongly recruited while observing actions during the learning of new motor patterns through imitation of other’s actions ([64]–[67] see also [68]). The same pMNS is also activated when participants simply view the actions of others without needing to replicate them, or when they simply execute these actions [23], [24]. Accordingly, it is thought that the pMNS transforms observed actions into motor codes required for the execution of similar actions. However, the role of the pMNS in the acquisition of arbitrary visuomotor associations, where it is critical to distinguish between rewarded (i.e. positive feedback) and unrewarded (i.e. negative feedback) actions in a particular context, remained unexplored. In our task, no novel motor patterns need to be acquired. Instead, novel associations need to be crafted between familiar motor patterns, stimuli and rewards. So far, we have focused on the fact that the processing of outcomes during visuomotor association learning shares neural substrates in our participants when performed by LeO and TE. Since LeO involves the observation of the actions of others during the stimulus presentation, and TE involves the execution of an action during the response phase, we suggest that the pMNS may be activated during the stimulus/response phase of each trial in our experiment. Given that previous action observation experiments describing the properties of the pMNS never distinguished correct from incorrect actions, it was unclear whether this system would also be recruited while our participants find out if the action was correct or not. Here, we therefore focused on analysing the outcome phase of each trial, and we found that both LeO and TE involved a brain network also active during simple action execution and observation and corresponding to the pMNS described in the literature. Interestingly, the BOLD activity in these areas presents an opposite pattern depending on whether the subjects were involved in a learning task or not (cf. Fig. 3B). The BOLD signal increase following outcome presentation was generally larger for observation (LeO) than for execution (TE). However, during the action observation/execution task, the signal was larger for execution (EXE) than for observation (OBS). The lesser activation in OBS compared to EXE is a common finding in the pMNS literature and is likely to be related to the fact that only about 10% of premotor neurons responds to action observation in primates [25], [69]. Why LeO has a slightly larger signal than TE in these somatosensory-motor regions is difficult to infer from our data, and we can only speculate about the origin of this effect. One possibility is that the BOLD signal in these somatosensorimotor regions is enhanced in LeO (compared to TE) as a consequence of the fact that in LeO (unlike in TE) the action was not executed by the participant during the SR phase, and that the participants may thus have a stronger urge to mentally re-enact the observed action upon finding out whether it was to be associated with the stimulus or not. In the absence of overt execution, this additional mental re-enactment of another’s action might be important to consolidate the stimulus-response link that needs to be established during our task.

Recent fMRI evidence from Gazzola and Keysers [23] and meta-analyses [24] showed that action observation and execution do not exclusively recruit the “classic” mirror areas (namely, the ventral premotor cortex and the inferior parietal lobule; see for example [68]), but additional brain areas such as the dorsal premotor cortex and the superior parietal lobule, as well as the supplementary and cingulate areas. Our results are in line with these findings, and suggest that the processing of outcomes during LeO and TE recruits regions involved in action execution and observation. While it is thus not surprising that pMNS regions are activated while participants move their hand in the TE condition and see others move in the LeO condition (the very definition of pMNS), we demonstrate that these regions activate while processing outcomes during LeO and TE, when no action was perceived or performed. This suggests that motor representations are activated twice during arbitrary stimulus-response-outcome associative learning. In TE, once when the participant executes a candidate action, and once when the participant finds out whether the action was successful or not during the acquisition phase. The latter activation becomes weaker in the consolidation phase, suggesting that reactivation of motor programs serves learning. During LeO, the first activation during action observation would represent a vicarious sharing of the attempted action, and resemble that often described in action observation experiments [24]. The second, however, would again serve learning, co-opting the mechanisms of TE learning by feeding it with vicarious rather than first-hand motor activations. In other words, we suggest that not only imitation learning (i.e. learn novel motor patterns through observation), but also abstract visuomotor associations learning-by-observation is partially supported by activation of the pMNS.

### Neural Systems Selectively Recruited During Learning-by-observation

Our study also revealed brain areas that are specifically activated during LeO. Whereas no brain activation was found to differentiate the processing of first correct outcomes across TE and LeO, the processing of others' errors showed significant differences across learning conditions. Brain areas emerged as significantly more activated during incorrect outcome presentation in observational *versus* individual learning. The activated clusters were localised bilaterally in the middle cingulate cortex and posterior medial frontal cortex (pMFC), the anterior insula and the posterior superior temporal sulci (pSTS) (Fig. 4B, Table 2B). Both pMFC and the anterior insula are thought to be components of the error-monitoring network [70]. The pMFC is located in the dorsal anterior cingulate cortex, which has been suggested to be involved in individual learning from errors [71], [72]. Current research indicates that the pMFC plays a crucial role in error-monitoring and subsequent behavioural adjustement [73]. In particular, a performance-monitoring system in the pMFC seems to signal the need for adjustments when action outcomes call for adaptations [74]. In addition, recent data from electrophysiological recordings in the monkey suggest that neurons in dorsomedial prefrontal selectively respond to another’s erroneous actions and that their activity is associated with a subsequent behavioral adjustment [75]. The anterior insular cortex is known to contribute to performance monitoring processes [70]. It has been proposed to be involved in autonomic responses to errors in non-social contexts [74] and to increase its activity with error awareness [76], [77]. This network has also been found to be active during error-detection in non-learning contexts [70], [73], [78] and its activity has not been found to differentiate others’ from individual’ errors [78]–[80] nor to depend on the experimental setting or social context [70]. Our results provide critical information about the role of the pMFC - anterior insula network in the processing of other's error during LeO. Research to date has identified an association between the magnitude of error-related activity and subsequent learning performance [77], [81], [82]. We speculate that the selective activity in the pMFC - anterior insula network may represent a neural correlate of the cognitive biases that psychology and neuroeconomics have described as the predisposition to process the errors of others differently than personal errors in humans. Among these, the ‘actor-observer’ cognitive bias consists in the tendency to attribute others’ failures to their personality, and one’s own failures to the situation [83]. Additional neuroimaging and behavioural research is needed to explore the relative effectiveness of individual and observational learning from others and individual errors (cf. [11], [84]).

Our study showed that the posterior superior temporal sulcus (pSTS) also specifically correlated with the processing of others' errors during learning-by-observation. Previous non-human primate connectivity data indicate that the STS is anatomically well situated to integrate information derived from both the ventral and dorsal visual pathways [85]–[87]. For this reason, several studies suggest that initial analysis of social cues occurs in the STS region, which is sensitive to stimuli that signal the actions of another individual. Particular attention was given to the posterior part of the STS, which has been characterized as the substrate of goal-driven action understanding [88] and social perception [89]. In general, current literature supports the idea that the perception of agency activates the pSTS [90] and that activity in pSTS may be part of a circuit associating observed actions with motor programs [91], [92]. In addition, the pSTS is thought to be involved in the attribution of mental states to other organisms [93]–[96] and the extraction of contextual and intentional cues from goal-directed behaviour [97]. Importantly, activity in pSTS has previously been found in humans during imitation of actions [98]. Our results show that this region is selectively activated during the processing of error signals early during observational learning. Therefore, our results are compatible with a role of pSTS in the processing of social cues, such as others' actions' outcomes, a necessary step during the early observational learning. In addition, the fact that the pSTS was more activated by the errors of others than self, could reflect more intensive mentalizing (what does the actor think now that he knows that this action didn’t work?) or a reactivation of the visual representation of the observed action in order to reduce its association with the stimulus.

## Conclusions

Our results suggest that the processing of others' outcomes during learning-by-observation shares a common brain network with trial-and-error learning. This network includes the dorsal fronto-parietal system, the associative fronto-striatal loop and the cerebellum. In addition, we showed that this shared network overlaps with the putative mirror neuron system, known to be involved during action observation and execution. This suggests that the pMNS, in addition to its role in acquiring new motor patterns during imitation learning, may mediate the vicarious learning of abstract visuomotor associations. Finally, we identified brain areas more activated for others- than self- errors during learning in the posterior medial frontal cortex (pMFC), the left anterior insula and the bilateral posterior superior temporal sulci (pSTS). We suggested that the pMFC and anterior insula, known to be crucial for error-detection, are involved in error monitoring during learning-by-observation. In parallel, the pSTS seems to provide information about social cues, such as others' actions' outcomes, a necessary step during the early phases of learning-by-observation. Overall, our study contributes to a better understanding of brain regions involved in vicariously learning stimulus-action-outcome associations by showing that this process recruits the mechanisms of the pMNS and the trial-and-error learning machinery.

## Supporting Information

Figure S1 **Brain networks commonly recruited during learning in both TE and LeO conditions.** Positive effect of the acquisition phase (i.e. incorrect outcomes +1st correct outcome), reflecting the common activations of TE and LeO during learning (t = 3.24, *p*unc<0.001; all clusters also survive *q*FDR<0.05). Clusters of activation are superimposed on to the average T1 image derived from all participants.
(TIFF)

Figure S2 **Brain networks commonly recruited during the acquisition phase of learning, action observation and execution.** The localizer t-map for the pMNS was inclusively intersected with the positive effect of the acquisition phase (see Fig.S1), reflecting the common activations of TE and LeO during learning (t = 3.24, *p*unc<0.001; all clusters also survive *q*FDR<0.05). Clusters of activation are superimposed on to the average T1 image derived from all participants.
(TIFF)

Table S1 Positive effect of the acquisition phase, reflecting the common activations of TE and LeO during learning (t = 3.24, *p*unc<0.001; all clusters also survive *q*FDR <0.05).
(DOC)

Table S2 Localizer t-map for the pMNS (t = 3.24, *p*unc <0.001).
(DOC)

Table S3 Intersection analysis between the localizer t-map for the pMNS and the positive effect of the acquisition phase, reflecting the common activations of TE and LeO during learning (t = 3.24, *p*unc <0.001; all clusters also survive *q*FDR<0.05).
(DOC)

Table S4 Interaction of correcteness (incorrect, 1^st^ correct) by learning condition (TE, LeO) (F1,14 = 12.06, *p*unc<0.001 but *q = *NS).
(DOC)
