# Supplementary figures and images for: Vicarious Neural Processing of Outcomes during Observational Learning
Source: PLoS One. 2013 Sep 5;8(9):e73879. doi: 10.1371/journal.pone.0073879 (PMC3764021; doi:10.1371/journal.pone.0073879)

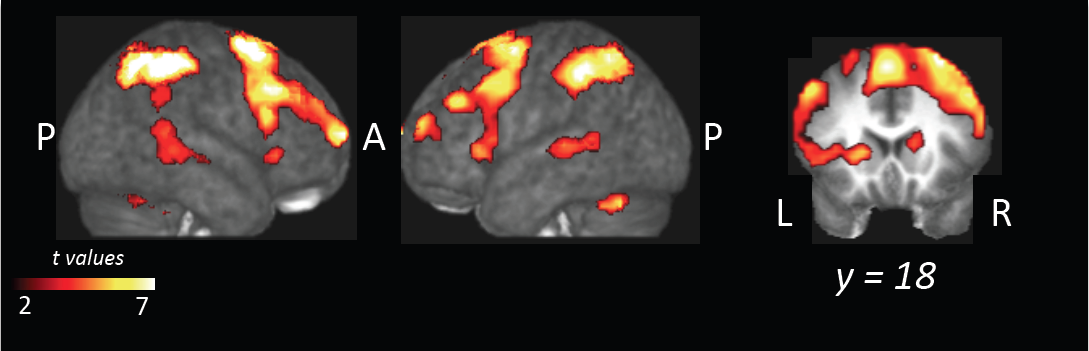

Supplement: Figure S1 — Brain networks commonly recruited during learning in both TE and LeO conditions. Positive effect of the acquisition phase (i.e. incorrect outcomes +1st correct outcome), reflecting the common activations of TE and LeO during learning (t = 3.24, punc<0.001; all clusters also survive qFDR<0.05). Clusters of activation are superimposed on to the average T1 image derived from all participants. (TIFF) [file pone.0073879.s001.tiff]

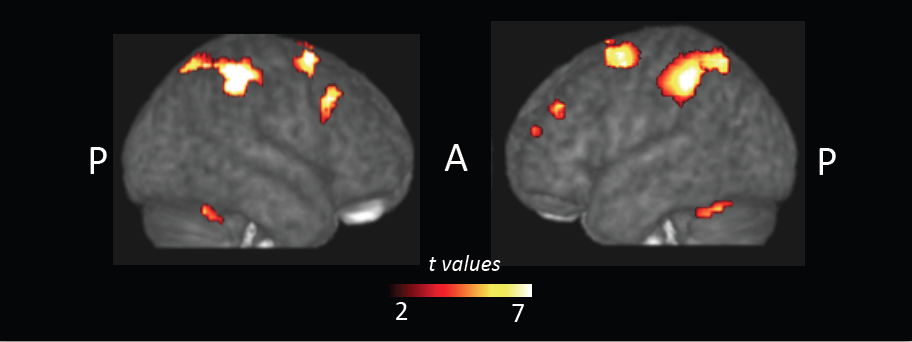

Supplement: Figure S2 — Brain networks commonly recruited during the acquisition phase of learning, action observation and execution. The localizer t-map for the pMNS was inclusively intersected with the positive effect of the acquisition phase (see Fig.S1), reflecting the common activations of TE and LeO during learning (t = 3.24, punc<0.001; all clusters also survive qFDR<0.05). Clusters of activation are superimposed on to the average T1 image derived from all participants. (TIFF) [file pone.0073879.s002.tiff]

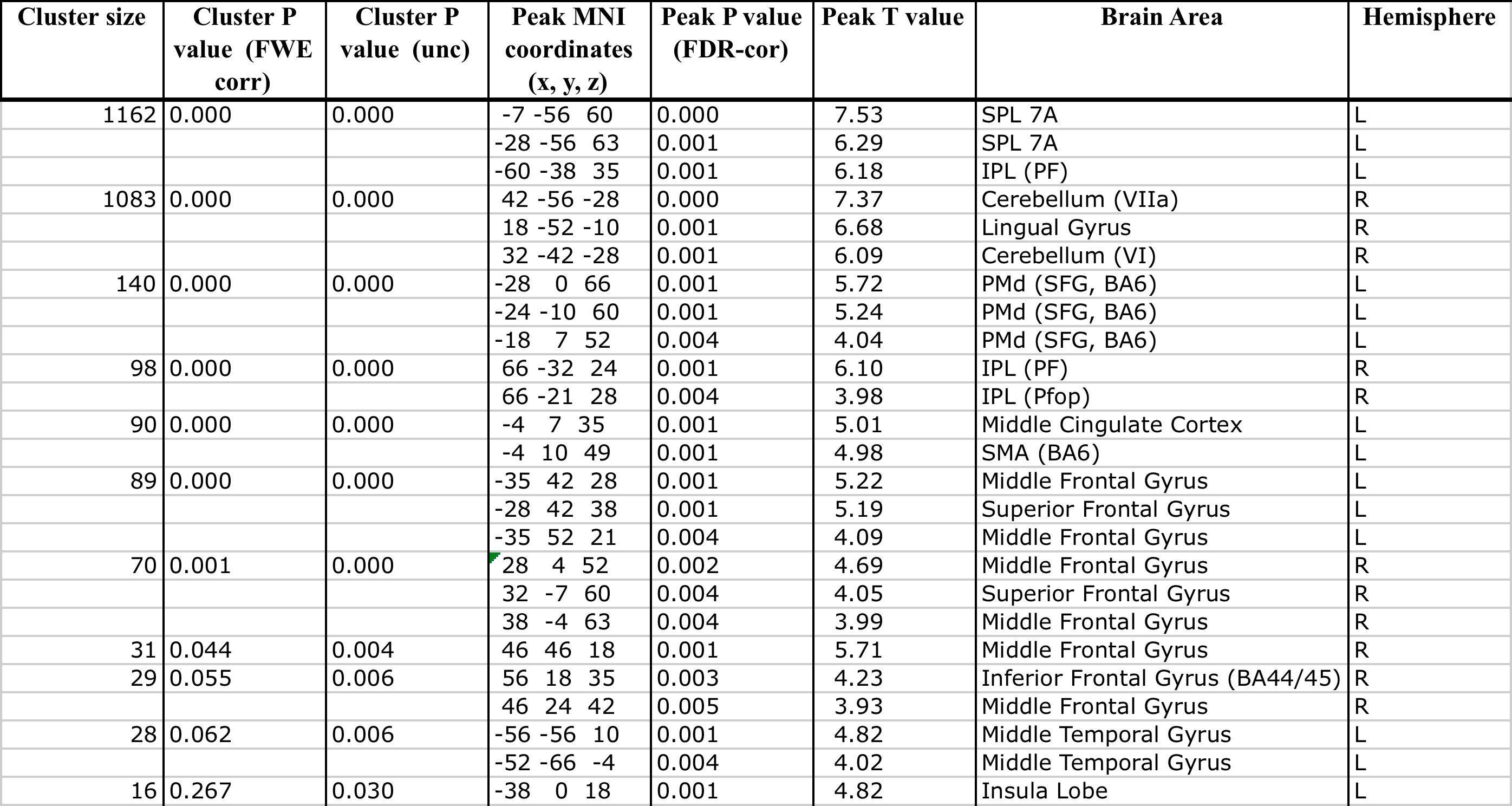


Abbreviations as in Table S1.

**Table S2.** Localizer t-map for the pMNS (t = 3.24, *p*unc < 0.001).

Supplement: Table S2 — Localizer t-map for the pMNS (t = 3.24, punc <0.001). (DOC) [file pone.0073879.s004.doc]
