# Supplementary material for: Vicarious Neural Processing of Outcomes during Observational Learning
Source: PLoS One. 2013 Sep 5;8(9):e73879. doi: 10.1371/journal.pone.0073879 (PMC3764021; doi:10.1371/journal.pone.0073879)
Supplement: Table S1 — Positive effect of the acquisition phase, reflecting the common activations of TE and LeO during learning (t = 3.24, punc<0.001; all clusters also survive qFDR <0.05). (DOC) [file pone.0073879.s003.doc]

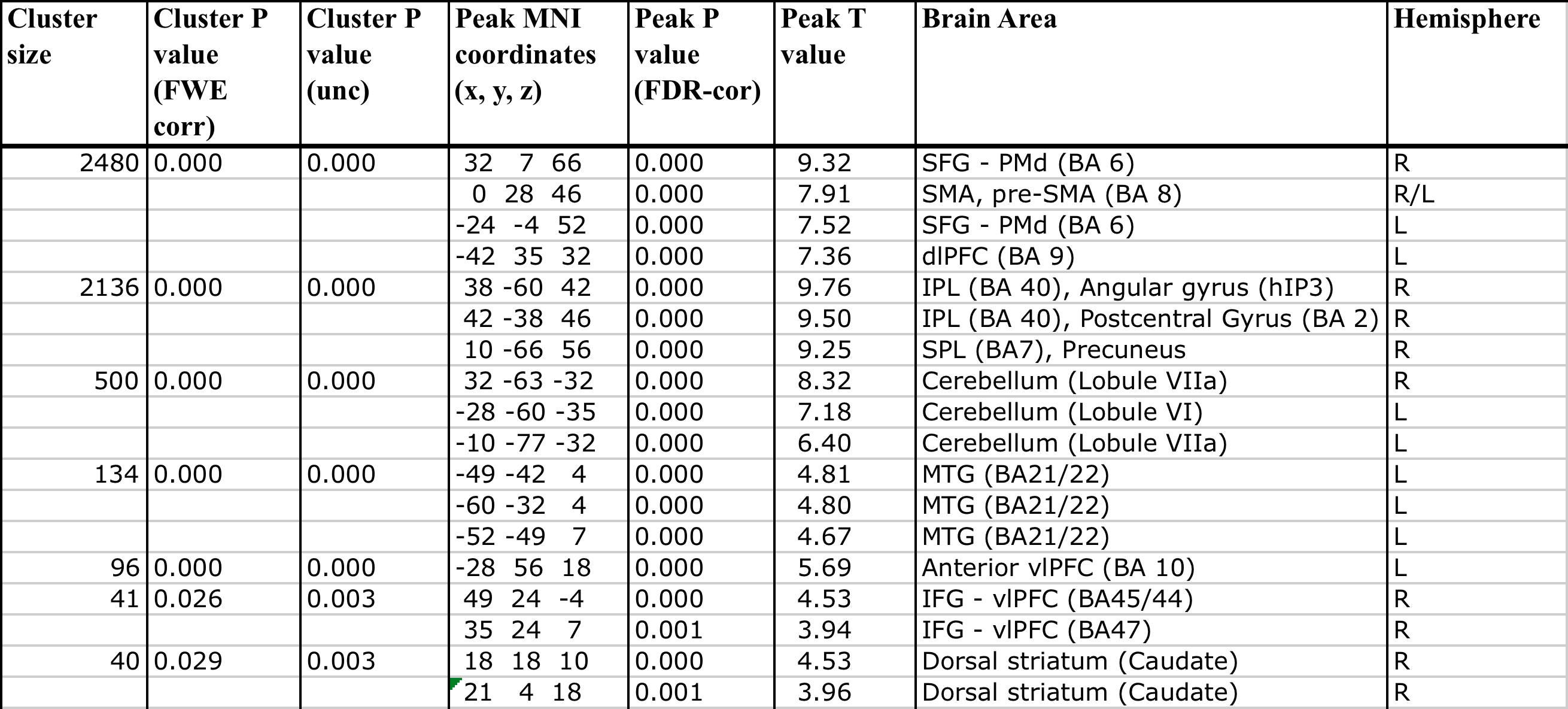


Abbreviations: IPL (inferior parietal lobule); SPL (superior parietal lobule); IFG (inferior frontal gyrus); SFG (superior frontal gyrus); MFG (middle frontal gyrus); IFG (inferior frontal gyrus); SMA (supplementary motor cortex); MTG (middle temporal gyrus); vlPFC (ventro-lateral prefrontal cortex); dlPFC (dorso-lateral prefrontal cortex); PMd (premotor dorsal); BA : Brodmann area.

**Table S1.** Positive effect of the acquisition phase, reflecting the common activations of TE and LeO during learning (t = 3.24, *p*unc<0.001; all clusters also survive *q*FDR < 0.05).
