# Supplementary material for: Vicarious Neural Processing of Outcomes during Observational Learning
Source: PLoS One. 2013 Sep 5;8(9):e73879. doi: 10.1371/journal.pone.0073879 (PMC3764021; doi:10.1371/journal.pone.0073879)
Supplement: Table S3 — Intersection analysis between the localizer t-map for the pMNS and the positive effect of the acquisition phase, reflecting the common activations of TE and LeO during learning (t = 3.24, punc <0.001; all clusters also survive qFDR<0.05). (DOC) [file pone.0073879.s005.doc]

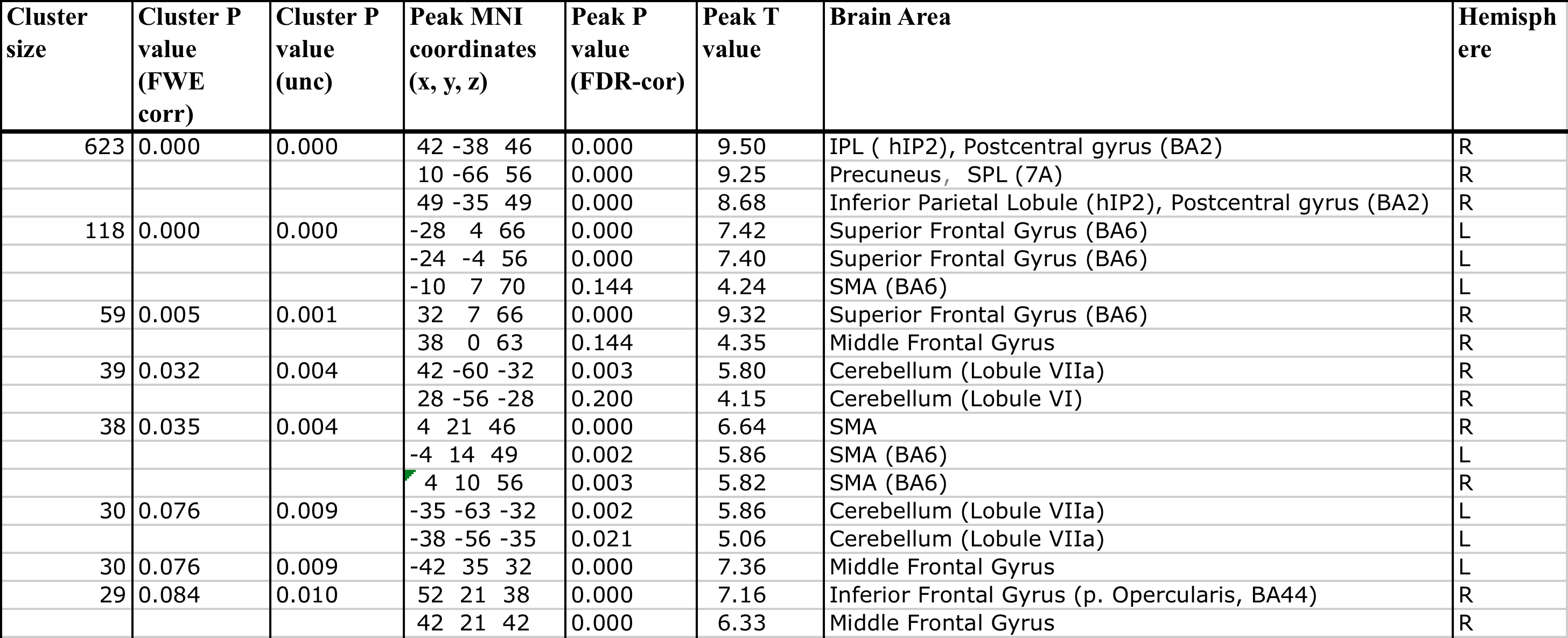
Abbreviations as in Table S1.

**Table S3.** Intersection analysis between the localizer t-map for the pMNS and the positive effect of the acquisition phase, reflecting the common activations of TE and LeO during learning (t = 3.24, *p*unc < 0.001; all clusters also survive *q*FDR<0.05).
