# Supplementary material for: Vicarious Neural Processing of Outcomes during Observational Learning
Source: PLoS One. 2013 Sep 5;8(9):e73879. doi: 10.1371/journal.pone.0073879 (PMC3764021; doi:10.1371/journal.pone.0073879)
Supplement: Table S4 — Interaction of correcteness (incorrect, 1st correct) by learning condition (TE, LeO) (F1,14 = 12.06, punc<0.001 but q = NS). (DOC) [file pone.0073879.s006.doc]

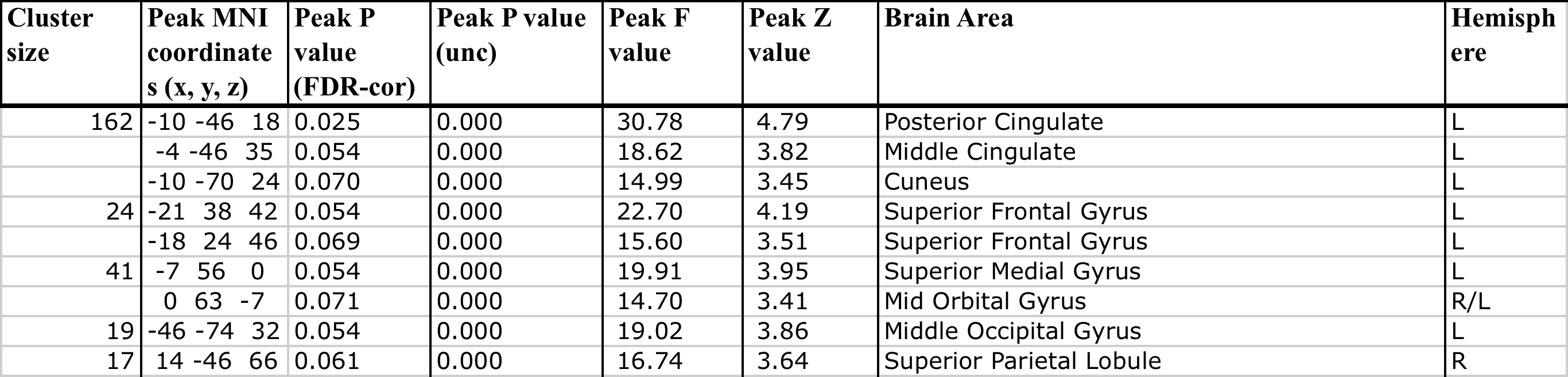


**Table S4.** Interaction of correcteness (incorrect, 1^st^ correct) by learning condition (TE, LeO) (F1,14=12.06, *p*unc<0.001 but *q=*NS).
